# DISOPRED3: precise disordered region predictions with annotated protein-binding activity

**DOI:** 10.1093/bioinformatics/btu744

**Published:** 2014-11-12

**Authors:** David T. Jones, Domenico Cozzetto

**Affiliations:** Bioinformatics Group, Department of Computer Science, University College London, Gower Street, London WC1E 6BT, UK

## Abstract

**Motivation:** A sizeable fraction of eukaryotic proteins contain intrinsically disordered regions (IDRs), which act in unfolded states or by undergoing transitions between structured and unstructured conformations. Over time, sequence-based classifiers of IDRs have become fairly accurate and currently a major challenge is linking IDRs to their biological roles from the molecular to the systems level.

**Results:** We describe DISOPRED3, which extends its predecessor with new modules to predict IDRs and protein-binding sites within them. Based on recent CASP evaluation results, DISOPRED3 can be regarded as state of the art in the identification of IDRs, and our self-assessment shows that it significantly improves over DISOPRED2 because its predictions are more specific across the whole board and more sensitive to IDRs longer than 20 amino acids. Predicted IDRs are annotated as protein binding through a novel SVM based classifier, which uses profile data and additional sequence-derived features. Based on benchmarking experiments with full cross-validation, we show that this predictor generates precise assignments of disordered protein binding regions and that it compares well with other publicly available tools.

**Availability and implementation:** http://bioinf.cs.ucl.ac.uk/disopred

**Contact:** d.t.jones@ucl.ac.uk

**Supplementary information:** Supplementary data are available at *Bioinformatics* online.

## 1 Introduction

Proteins experience more or less pronounced structural changes while performing their functions. In particular, completely unfolded states or transitions between structured and unstructured conformations are distinguishing features, which enable the physiological activities of intrinsically disordered proteins (IDPs) and intrinsically disordered regions (IDRs). Our understanding of the biological importance and of the widespread occurrence of this phenomenon is gradually expanding, thanks to the fine-tuning of experimental techniques and to computational genome-wide surveys. The DisProt database (Sickmeier *et al*., 2007) stores manually curated data for IDRs, but annotations are accumulating at a very slow rate: DisProt v. 6.02 reports 1539 IDRs and ∼40% of them still have unknown function. Therefore, bioinformatics plays a major role in researching the occurrence, the biological function, and the involvement of intrinsic disorder in phenotype and disease.

Many computational methods can predict IDRs within protein sequences (Deng *et al.*, 2012; Orosz and Ovadi, 2011). Propensity-based approaches were inspired by the observed enrichment of polar and charged amino acids and by the under-representation of hydrophobic and aromatic residues in IDRs (Dosztanyi *et al.*, 2005; Linding *et al.*, 2003; Prilusky *et al.*, 2005). Further comparative analyses also highlighted differences in the patterns of length, location and sequence conservation among IDRs, and machine-learning techniques have been trained to detect them (Eickholt and Cheng 2013; Hirose *et al.*, 2007; Ishida and Kinoshita 2007; Liu and Rost 2003; Shimizu *et al.*, 2007a,b; Ward *et al.*, 2004). Finally, meta-predictors integrate the output of independent tools through an array of algorithms (Ishida and Kinoshita 2008; Kozlowski and Bujnicki 2012; Schlessinger *et al.*, 2009).

Objective benchmarking of protein disorder prediction methods is essential before these can be used for further inferences. CASP (Critical Assessment of techniques for protein Structure Prediction) has been testing these predictors since 2002 (Melamud and Moult, 2003) and has tracked moderate progress since. Current prediction accuracy for IDRs spanning up to 30 residues is estimated at roughly 70% (Monastyrskyy *et al.*, 2014) and this has already allowed us to investigate the role that protein disorder plays in living organisms at different levels of complexity (Cozzetto and Jones, 2013)—including the organization and the re-wiring of protein–protein interaction networks, cellular differentiation and development and human disease (Buljan *et al.*, 2013; Perkins *et al.*, 2010).

At the molecular level, it is important to grasp whether IDRs act as flexible linkers or have binding activity and, if so, what molecules they recognize. ANCHOR was the first program to tackle the identification of disordered protein-binding regions (Meszaros *et al.*, 2009) using a linear regression model of the estimated stabilizing energy that IDRs would gain by folding upon binding. The statistical model was learnt from a small set of chains with evidence for being unstructured in isolation, but structured in complex with their partners. A few machine learning approaches have been parameterized on larger sets of solved protein-peptide complexes, where the peptides are likely to be disordered in the unbound state. MoRFpred (Disfani *et al.*, 2012) combines annotation transfers by similarity to the output of a support vector machine (SVM) that examines sequence conservation data, amino-acid physicochemical properties and predictions of intrinsic disorder, relative solvent accessibility and residue flexibility. MFSPSSMpred (Fang *et al.*, 2013) considers only sequence profiles, which are pre-processed to enhance the signal of local conservation within the fast evolving landscape of IDR sequences. Finally, PepBindPred (Khan *et al.*, 2013) first attempts to estimate the binding affinity of tripeptides from the input sequence to a library of known protein-binding domains, and then feeds these data to a bidirectional recurrent neural network along with additional features.

Here we describe the latest incarnation of DISOPRED, which was originally trained on evolutionarily conserved sequence features of IDRs from missing residues in high-resolution X-ray structures. DISOPRED3 extends the previous architecture with two independent predictors of intrinsic disorder, one module that combines the intermediate results and one component that annotates protein-binding IDRs. During the CASP9 and CASP10 experiments aimed at monitoring the state of the art in IDR prediction from sequence, the assessors ranked the updated server at the top or near the top across a range of evaluation measures and test cases (Monastyrskyy *et al.*, 2011, 2014). Using the same data, we demonstrate that DISOPRED3 is more specific than its predecessor and that it produces more accurate predictions across different IDR lengths and positions along the test sequences.

Residues in the predicted IDRs are annotated as folding upon protein binding through a new SVM trained to distinguish short peptides bound to globular domains from unbound protein domain linkers using sequence-derived features. Through stringent cross-validation experiments, we show that this predictor can generate precise annotations and that it compares well with ANCHOR, MoRFpred and MFSPSSMpred based on a carefully assembled test set derived from database annotations and scientific reports.

## 2 Methods

### 2.1 Datasets for protein disorder prediction

The new components aimed at IDR detection were trained with a concatenation of two datasets. The first was all entries (228 in total) from the Disprot v5.0 database (Sickmeier *et al.*, 2007) flagged as being derived from either NMR or biophysical methods. The other dataset was a redundancy-reduced (percentage sequence identity <90%) subset of high resolution (≤2.2 Å) X-ray structure chains from PDB (Berman *et al.*, 2000) derived from PISCES (Wang and Dunbrack, 2005) compiled in February 2010. Chains shorter than 25 amino acids were discarded. Missing residues, including those with occupancy equal to zero, were treated as disordered.

Position-specific scoring matrix (PSSM) scores were calculated for each residue using three iterations of PSI-BLAST (Altschul *et al.*, 1997) running on the UniRef90 data bank (Suzek *et al.*, 2007) with an inclusion *E*-value threshold of *h* = 0.001.

DISOPRED3 was registered as a server at CASP9 and CASP10—group ids 015 and 170, respectively—and made predictions for all assessed targets. The corresponding predictions are therefore available at the Prediction Center website; DISOPRED2 predictions for the same protein sets were generated as a prerequisite and stored locally.

The reference classification of the residues as ordered or disordered was taken from the Prediction Center website and was based on the structural data available before the final meetings. The amino acids were regarded as disordered if and only if either they were not assigned spatial coordinates, or the positions of their Cα atoms were more than 3.5 Å away across different chains or NMR models in the LGA (Zemla, 2003) structural alignment.

### 2.2 Datasets for protein-binding site prediction

A set of 840 peptides, with lengths between 5 and 25 amino acids and solved in complex with globular protein domains, was initially obtained from a previous study (Disfani *et al.*, 2012). We discarded 196 peptides that couldn’t be mapped onto UniProtKB (UniProt, 2014) sequences with SIFTS (Velankar *et al.*, 2013)—because they were synthetic constructs, or the PDB files had been superseded, or the ATOM records mapped onto discontinuous fragments. We also removed 247 chains sharing 30% or more sequence identity to other regions, based on the BLASTP alignments generated with the recommended settings for short peptides (-seg no -matrix PAM30 -gapopen 10 -gapextend 1 -word_size 3). The positive training set was therefore made up of 5501 amino acids from 397 regions occurring in as many PDB chains (372 UniprotKB sequences). Just 104 of these UniProtKB chains were also used as positive examples for the new disordered residue prediction modules. This limited overlap mostly reflects the different length requirements for inclusion into the two training sets.

Negative training examples were obtained from unbound protein domain linker regions in known protein structures. A total of 1164 linkers annotated in CATH v.3.5 (Sillitoe *et al.*, 2013) and spanning between 5 and 60 amino acids were screened for the lack of interactions with other molecules in the same PDB file. Contacts with other protein and nucleic acid chains were identified when any two heavy atoms were closer than 6 Å; for ligands and metal ions the distance threshold was set to 3.9 Å. As for the positive instances, we discarded the linkers that didn’t map completely to UniProtKB sequences with SIFTS or with at least 30% sequence identity to other regions. The negative training set consisted of 4930 amino acids from 373 protein domain linkers occurring in 322 PDB chains (297 UniprotKB sequences).

To build an independent benchmark set, we thoroughly mined database annotations and scientific reports to collate 29 protein chains, which have been investigated using biophysical techniques and have been shown to be disordered in isolation and to fold upon protein binding. These sequences include 4077 disordered residues forming 36 regions, within which 37 protein-binding sites occur spanning between 5 and 47 positions and comprising a total of 708 amino acids. Supplementary Table S1 reports detailed information about such test proteins, the annotated IDRs and segments undergoing disorder-to-order transitions as they bind other proteins.

### 2.3 DISOPRED3 neural networks

The design goal for the third version of DISOPRED was to counter the tendency for DISOPRED2 to under-predict long disordered regions. This was very clear from the independent assessment carried out in CASP8 (Noivirt-Brik *et al.*, 2009). Although the DISOPRED2 SVM offers adjustable specificity, and so allows some reduction of under-prediction as a whole, we were unable to tune it to specifically improve long-region prediction. We surmise this is due to the lack of long disordered regions in the X-ray-derived training sets used to train DISOPRED2. To try to tackle this issue, we opted to return to the original neural network based DISOPRED method (Jones and Ward, 2003), but trained on data rich in long disordered regions (see Section 2.1). Although we explored SVM classifiers for long-region prediction, we found the neural network method performed better on this particular problem. The neural network architecture, feature set and training procedures were as previously described.

A small second-stage neural network was then trained to combine the three component predictors into a single prediction output.

This small network comprises 15 × 4 inputs, 15 hidden units and 2 output units (ordered/disordered). The 15 × 4 inputs represents a window of 15 positions centered around the residue being classified, with 3 outputs from the first-level component methods (SVM, neural network and nearest neighbor classifier) plus a fourth input per position to indicate missing data (i.e. where the window extends beyond the N or C-terminus). Prediction confidence is estimated by considering the difference between the two outputs (disordered–ordered), which effectively compensates for the effects of unbalanced training, and thus requires no further calibration for the output difference to be considered directly as a posterior probability.

### 2.4 DISOPRED3 nearest neighbor classifier

In addition to the DISOPRED2 SVM and the long-region neural network predictors, we decided to add a third predictor, namely a nearest neighbor classifier. This was done for practical reasons, because a nearest neighbor classifier needs no training, and so it is easy to incorporate very up-to-date data in the prediction process, whereas the other classifiers require a great deal of time and effort to train on new data. The addition of the nearest neighbor classifier thus makes DISOPRED3 more easily updated and maintained.

Nearest neighbor classification was carried out by comparing every window of seven residues in the target protein with every seven-residue window taken from the profiles in the reference set. This comparison is made on the basis of total PSSM score, and the order/disorder label for each residue is decided by transferring the labels from the reference protein windows that have the highest match scores. In most cases, the label arises from considering the maximum of 7 scores, as aside from the residues near the termini, most residues end up contributing to the match scores in seven different offset positions.

### 2.5 Training procedures for protein-binding site prediction

To determine residues which might be part of protein-binding sites within disordered regions, we employed an SVM classifier rather than a neural network, as the data set was known to be extremely biased towards negative cases. Using a sliding window of size 15, we derived three independent SVM classifiers from the training data that are based on (i) single sequences alone, where each amino acid was encoded by the similarity values in the corresponding entry in the Blosum62 matrix; (ii) the PSSM values obtained after three search iterations of PSI-BLAST against UniRef90 with a profile-inclusion threshold of *h* = 0.001; (iii) the same PSSM scores, followed by the length of input region, the start and the end positions of the region relative to the whole protein chain, a flag for windows extending beyond the protein termini and the amino acid composition of the window under consideration. The PSSM scores were linearly scaled to [0.0, 1.0] based on the maximum and minimum values observed for each amino acid in the whole training set, while for protein sequence length the natural logarithmic scale was adopted.

To perform proper cross-validation, separate training runs were carried out for each of the 29 test proteins. During training, we excluded from the training data any sequence found to be similar to the target chain. We used a sequence identity threshold of 25% in the case of the SVM classifier based on sequence data alone and a PSI-BLAST *E*-value cut-off of 0.001 for all profile-based predictors. We ran LIBSVM (Chang and Lin, 2011) with a radial basis function kernel to identify the trade-off and gamma parameters that give the highest average Matthews correlation coefficient (MCC)—see below for the definition—across 10-fold cross validation experiments. The imbalance between the positive and negative classes was addressed by setting the cost parameter of the positive class to the ratio of negative to positive examples used for training at each iteration. The resulting parameters were then passed to LIBSVM to learn a regression model from the whole training set.

### 2.6 Evaluation measures

Performance of binary classification was evaluated based on standard measures, including sensitivity (also known as recall), specificity, precision, the MCC, and *F*_1_ score

(1) $$\text{Sensitivity}=\frac{\text{TP}}{\text{TP}+\text{FN}}$$

(2) $$\text{Specificity}=\frac{\text{TN}}{\text{TN}+\text{FP}}$$

(3) $$\text{Precision}=\frac{\text{TP}}{\text{TP}+\text{FP}}$$

(4) $$\text{MCC}=\frac{\left(\text{TP}\cdot \text{TN}\right)-\left(\text{FP}\cdot \text{FN}\right)}{\sqrt{\left(\text{TP}+\text{FP}\right)\cdot \left(\text{TP}+\text{FN}\right)\cdot \left(\text{TN}+\text{FP}\right)\cdot \left(\text{TN}+\text{FN}\right)}}$$

(5) $$F_{1}=2\cdot \frac{\text{precision}\cdot \text{recall}}{\text{precision}+\text{recall}}$$

where TP is the number of residues correctly labelled as positives (true positives); TN is the number of residues correctly labelled as negatives (true negatives); FP is the number of misclassified negative cases (false positives); and FN is the number of misclassified positive cases (false negatives).

For disordered residue predictions, ROC analysis was carried out in the light of the output probability scores. For each value *v*∈[0.0, 1.0], we considered as putatively disordered those and only those residues with a score higher than or equal to *v*, and as putatively ordered the remaining ones. Such binary classifications helped calculate TP, FP, FN, TN, sensitivity and specificity. Finally, ROC curves were generated from the pairs (1-specificity, sensitivity) corresponding to decreasing values of *v* and the area under the curve (AUC) was calculated with the trapezoid integration method in the pROC package (Robin *et al.*, 2011) for R (R Core Team, 2012).

In the statistical comparisons of method performance, we always test the null hypothesis that DISOPRED3 is not more accurate than DISOPRED2 against the alternative hypothesis that DISOPRED3 outperforms DISOPRED2. For binary classifications, we carried out 10^5^ resampling experiments: each time we randomly sampled 80% of the proteins, we estimated the scores for the two predictors, and we finally recorded the values of the paired differences. *P*-values for the null hypotheses were estimated by comparing such differences with zero. For probability-based predictions, we assessed the statistical significance of the differences in AUC values with the DeLong non-parametric test (DeLong *et al.*, 1988) as implemented in the pROC package.

## 3 Results and discussion

DISOPRED3 participated as a server at the CASP9 and CASP10 experiments, and the official assessment reports acknowledged its value by ranking it at the top or near the top across a number of tests (Monastyrskyy *et al.*, 2011, 2014). Although not exhaustive, the list of predictors tested at CASP is nonetheless representative of the latest developments in the field, which are also usually publicly available to the scientific community. Therefore, the results and discussion below focus on the comparison of DISOPRED3 with its predecessor using standard evaluation measures, and then on its use for protein-binding region detection.

### 3.1 Improvements over DISOPRED2

**Fig. 1.**
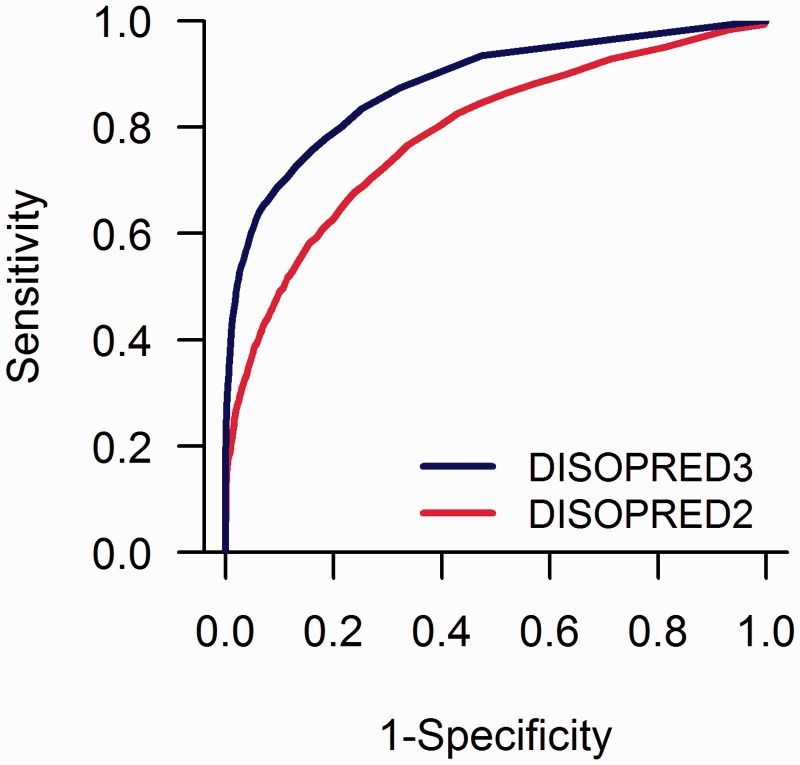
ROC curves of probability-based IDR predictions for DISOPRED3 and DISOPRED2 on the CASP10 data

The analysis of both the binary and the probability-based classifications against the CASP10 reference assignments is summarized in Figure 1, Tables 1 and 2. The evaluation results based on CASP9 data are consistent with those presented here and are outlined in Supplementary Tables S2 and S3. Overall, our self-assessment shows that DISOPRED3 greatly reduces the number of false positives with negligible loss of sensitivity. This leads to a statistically significant increase in precision (*P* < 1e-5), MCC (*P* < 1e-5) and AUC (*P* < 2.2e-16), which are known to correlate with the under-prediction of the minority class in the context of *imbalanced data* classifications. On the other hand, the observed difference in terms of balanced accuracy defined as the average of sensitivity and specificity, is unlikely to be significant (*P* = 0.06), because this measure is generally affected by the over prediction of the minority class.

**Table 1.** Comparison of DISOPRED3 and DISOPRED2 performance divided into IDR length ranges

Measure	No IDR shorter than 4 aas		No IDR shorter than 20 aas	
	DISOPRED2	DISOPRED3	DISOPRED2	DISOPRED3
Sensitivity	0.396	0.384	0.343	0.533
Specificity	0.941	0.991	0.941	0.991
Precision	0.323	0.755	0.134	0.616
MCC	0.307	0.517	0.181	0.563
AUC	0.787	0.880	0.731	0.904

	No IDR shorter than 30 aas		No IDR shorter than 40 aas	

Sensitivity	0.419	0.581	0.340	0.319
Specificity	0.941	0.991	0.941	0.991
Precision	0.084	0.459	0.024	0.131
MCC	0.166	0.509	0.076	0.199
AUC	0.745	0.900	0.726	0.891

**Table 2.** Performance comparison between DISOPRED releases by IDR position along sequences

Measure	Terminal protein regions		Internal protein regions	
	DISOPRED2	DISOPRED3	DISOPRED2	DISOPRED3
Sensitivity	0.665	0.584	0.275	0.293
Specificity	0.825	0.914	0.947	0.995
Precision	0.645	0.766	0.209	0.745
MCC	0.486	0.541	0.195	0.452
AUC	0.825	0.877	0.755	0.850

The usefulness of the additional modules for intrinsic disorder prediction becomes clear when the focus shifts to increasingly longer IDRs. It's fair to say that the CASP target selection procedure is mainly aimed at releasing protein sequences, the structures of which are unknown but expected to be solved by the end of the 3-month prediction stage or soon after that. This inevitably selects against disordered residues—and long IDRs in particular—which can hinder structure determination efforts. In fact, ∼60% of the disordered residues in the benchmark set occur in IDRs shorter than 20 amino acids. With this caveat in mind, we looked at a few length ranges, by masking both the reference and predicted state assignments for IDRs shorter than the selected thresholds. Table 1 reports the values for the recalculated evaluation measures after IDRs shorter than 20, 30 and 40 residues were discarded. As the number of negative cases didn’t differ, the corresponding specificity values were unchanged.

We observe a clear improvement over DISOPRED2 across the whole board. In particular, the higher levels of sensitivity for segments with at least 20 or 30 amino acids clearly confirm the added value of the neural network and of the nearest neighbor classifier of long IDRs. Certainly, a more balanced test set would allow for more detailed investigations, but the occurrence of 307 and 197 disordered residues respectively in such subsets is enough for such a conclusion. For regions of 40 or more residues, DISOPRED3 still outperforms the previous release, but the rather limited number of positive test cases—just 94 residues in two distinct stretches—makes it hard to draw general and sound conclusions.

We finally compared the prediction accuracy of the two DISOPRED versions on the subsets of terminal and internal IDRs. Here, terminal amino acids are those within 10 positions of each protein sequence termini, while all the remaining ones are regarded as internal. The higher abundance of IDRs at protein’s N- and C-termini is a well-known bias, and represents one of the helpful features exploited by machine learning methods. Correct identification of IDRs far from termini is therefore expected to be more challenging.

Generally, the outcome of this test—shown in Table 2—mirrors the findings based on the whole dataset, with DISOPRED3 giving more consistent results. For amino acids further than 10 positions from the protein’s N- or C-terminus, we also observe that DISOPRED3 makes a slight improvement in sensitivity over DISOPRED2, which is likely correlated to the higher occurrence of long IDRs at internal positions.

### 3.2 Effectiveness of disordered protein-binding region predictions

Over the past few years, tentative steps have been taken towards annotating the molecular activities of predicted IDRs, including the identification of relatively short regions that fold upon interacting transiently with other proteins. We initially set out to train an SVM-based classifier for this task using a 15 amino acid long sliding window, which considers sequence profile data, the length and location of the input IDR relative to the whole protein sequence, and the amino acid composition of the window. The length and position of IDRs, indeed, correlate with general protein functional categories and so can be useful for the annotation of proteins with distant or no homologues with known function at all (Lobley *et al.*, 2007; Minneci *et al.*, 2013). In particular, regions closer to the chain’s termini are very likely to perform binding activities while those further away could also act as flexible linkers.

Despite the availability of many protein disorder predictors based on evolutionary information, there is still no unanimous consensus in the field about the extent of usefulness offered by sequence profile data. This prompted us to investigate the possibility of learning the classification of disordered protein-binding regions from sequence data alone, from PSSM values, and from the list of features mentioned above. For this purpose, we tested and compared with full cross-validation these SVM-based predictors against a set of 37 protein regions shown to be disordered in isolation and folded in complex with other globular protein domains using biophysical techniques—mostly NMR spectroscopy. Here, we measure prediction accuracy conservatively: all disordered residues not annotated as protein binding were considered as not being able to do so, even under different physiological conditions—e.g. in the presence of other proteins. We certainly appreciate that these assumptions are highly conservative and arguably somewhat unrealistic, given the occurrence of IDRs in protein–protein interaction network hubs. However, at this point in time this is the only viable strategy that we can envisage to penalize and avoid over predictions, and indeed it appears to be the approach used in the similar areas, such as the assessment of methods for Gene Ontology terms or post-translational modification predictions.

The summary evaluation results in Table 3 show that the use of single sequence information provides less sensitivity but more specificity than the use of PSSM scoring. Overall, PSSM data would appear to provide slightly better predictions, as gathered from the precision, *F*_1_ and MCC scores. Adding information about IDR length, location and amino acid composition greatly boosts specificity and MCC, at the expense of sensitivity. Given the correct residue level assignments to the disordered regions, these methods strike rather different balances between sensitivity and specificity, but overall they appear to be useful, as they outperform naïve approaches that randomly label each disordered residue as protein binding or not.

**Table 3.** Benchmark of SVM classifiers of disordered protein-binding residues trained on different sets of sequence-derived features

Measure	Naïve	Sequence	PSSM	PSSM, IDR location, length and AA composition
Sensitivity	0.500	0.640	0.781	0.270
Specificity	0.500	0.517	0.401	0.940
Precision	0.174	0.218	0.215	0.485
MCC	0.000	0.119	0.143	0.269
*F*_1_	0.258	0.325	0.337	0.347

Naïve predictions correspond to random labelling of the known disordered residues as folding upon protein binding or not with equal probability.

### 3.3 Comparison with other tools detecting disordered protein-binding regions

The performance of the predictor based on profile data, IDR location and length and window composition was also compared with publicly available tools and with a naïve approach that randomly labels the target sequence amino acids as either disordered protein binding or not with equal probability. The three other programs in the benchmark are ANCHOR (Meszaros *et al.*, 2009), MoRFpred (Disfani *et al.*, 2012) and MFSPSSMpred (Fang *et al.*, 2013), which are available for download or online use; PepBindPred (Khan *et al.*, 2013) was not tested, given the running times needed for molecular dynamics simulations.

To reduce biases on the one hand and to keep a reasonable number of test cases on the other, we ended up limited to considering only 9 of the 29 protein chains we originally collected, because the remaining chains belong to the published training sets of ANCHOR, MoRFpred or MFSPSSMPred. Because we could not ensure full cross-validation of these tools, the accuracies reported below may be overestimated. On the other hand, DISOPRED3 predictions were generated using the appropriate fully cross-validated SVMs and the disordered residue assignments predicted from sequence—see Supplementary Table S4 for details about IDR prediction accuracy. All reference annotations and predictions collected for this study are available from our own website, so that readers can easily reproduce our results or compare their own methods.

Again, prediction accuracy was measured conservatively, by considering each residue as disordered protein binding if and only if there is clear experimental evidence from biophysical methods. Prediction accuracy was gauged using precision and recall analysis as well as MCC scores, though the former approach might be more appropriate to keep the focus on the task of IDR functional annotation rather than on the problem of their identification. The assessment would otherwise be inevitably affected by the vast majority of ordered residues in the negative set and by the difficulty of identifying disordered residues never interacting with other proteins.

Table 4 shows that, overall, DISOPRED3 performs well when compared with all the other approaches, by providing the most specific and precise predictions of residues that undergo disorder-to-order transitions upon protein binding. The benefits of the SVM for protein-binding region annotation is demonstrated by the massive reduction in the number of false positive assignments that would be made by tagging all predicted disordered residues as protein binding (method ‘DISOPRED3 no DPB SVM’). This supports the usefulness of unbound domain linker regions to represent IDRs that do not bind proteins. The other two machine-learning based methods, MoRFpred and MFSPSSMpred, achieve nearly identical *F*_1_ scores to DISOPRED3 due to their ability to recall slightly more disordered protein-binding residues. Finally, ANCHOR attains the highest level of sensitivity, though at the heavy expense of lower specificity. However, due to the limited number of IDRs that fold upon protein binding in this test set, it is difficult to make statistically sound conclusions about differences in method performance. Certainly, we hope that larger corpuses of confirmed protein annotations, along with side-by-side blind testing experiments will help address this point in the future

**Table 4.** Benchmark results of DISOPRED3 against other approaches for disordered protein-binding prediction

Method	Sensitivity	Specificity	Precision	*F*_1_	MCC
DISOPRED3	0.147	0.958	0.218	0.176	0.126
MoRFpred	0.190	0.922	0.162	0.175	0.104
MFSPSSMpred	0.206	0.900	0.152	0.175	0.093
Naïve	0.500	0.500	0.074	0.129	0.000
DISOPRED3 no DPB SVM	0.307	0.613	0.059	0.100	−0.043
ANCHOR	0.288	0.536	0.047	0.081	−0.092

DISOPRED3 no DPB SVM is a baseline method that considers all disordered residues identified by DISOPRED3 as involved in protein binding.

We further investigated whether the false positive assignments of each program were sufficiently close to the validated regions, that users with prior knowledge about the test proteins might still gain some benefit from these predictions after manual inspection. To this end, we focused on those amino acids predicted as disordered protein binding but observed in complex with no other protein domain. For every such residue, we initially calculated the minimum sequence separation from the closest amino acid annotated as a positive instance. We then computed the proportion of false positive predictions within 5, 10, 20 and 35 positions of the validated sites. This largest distance was determined as half the length of the shortest protein sequence in the test set.

The numerical results of this study are reported in Supplementary Table S5 and show that ∼30% of the false positive predictions are no more than 35 positions away from the binding sites so far characterized. Because the structural, dynamic and functional aspects of these proteins have not been sufficiently characterized, it is difficult to tell whether these predictions are incorrect or represent plausible hypotheses awaiting better experimental verification.

Overall, the above results show substantial room for improvement in different areas. Certainly, more sensitive IDR predictions would allow for the detection of additional protein binding disordered sites. As far as DISOPRED3 is concerned, the current results show that most false negative assignments correspond to residues predicted as ordered in the first place. In the future, we are keen to investigate more effective integrative approaches of the prediction modules for IDR detection and protein-binding site annotation. Further improvements will definitely stem from the availability of large sets of residue-level annotations of IDRs and of their biological roles that can be automatically parsed. Many DisProt entries contain IDRs undoubtedly involved in protein–protein interactions, but often the resolution of the available experimental data is not sufficient to locate the protein-binding sites accurately. In other cases, manual annotation efforts apparently do not follow standard guidelines—an issue exacerbated by the lack in the database schema of attributes for ligand binding sites. Consequently, long protein-binding IDRs are sometimes reported, but the specific binding residues documented in the cited primary literature are not—compare the entry DP00608 and the referenced article (Wang *et al.*, 2010), for instance.

## 4 Conclusion

We have documented the DISOPRED3 program for protein disorder prediction and for protein-binding site annotation within disordered regions. The tool first identifies disordered residues through a consensus of the output generated by DISOPRED2 and two additional machine-learning based modules trained on large IDRs, and then annotates them as protein binding through an additional SVM classifier. The intrinsic disorder predictor attains fairly high levels of prediction accuracy across different test conditions, and can be regarded as state of the art—as independently reported by the CASP9 and CASP10 assessment teams—thus confirming the usefulness of integrating multiple complementary approaches. Compared with its predecessor, DISOPRED3 shows improved sensitivity for IDRs with twenty or more amino acids, as well as increased overall specificity. Such predictions form a useful basis for the identification of protein-binding regions mediating key transient interactions through a novel SVM, which compares well with existing tools for the same task.

On these grounds, DISOPRED3 is expected to represent a useful addition to the toolbox for the functional annotation of proteins and proteomes. Notwithstanding, further advances are needed to enhance the sensitivity of IDR detection and of the protein-binding sites therein. Of course, the accumulation of additional experimental information and its availability in a structured, easy-to-parse manner will be beneficial—as already witnessed in other areas of computational biology. However, new ideas will be crucial to make major progress, especially in the attempt to link the binary classifications of ordered and disordered residues to biological functions at different scales of complexity. In particular, initial system-level investigations of the role of protein disorder in cellular development and differentiation have already begun thanks to the increasing availability of genome-wide heterogeneous datasets. In this context, the design and implementation of reliable and more scalable tools will be of special relevance.

## Supplementary Material

Supplementary Data

## References

[btu744-B1] Altschul S.F.*.* (1997) Gapped BLAST and PSI-BLAST: a new generation of protein database search programs. Nucleic Acids Res., 25, 3389–3402. 9254694 10.1093/nar/25.17.3389 PMC146917

[btu744-B2] Berman H.M.*.* (2000) The Protein Data Bank. Nucleic Acids Res., 28, 235–242. 10592235 10.1093/nar/28.1.235 PMC102472

[btu744-B3] Buljan M.*.* (2013) Alternative splicing of intrinsically disordered regions and rewiring of protein interactions. Curr. Opin. Struct. Biol., 23, 443–450. 23706950 10.1016/j.sbi.2013.03.006

[btu744-B4] Chang C.-C. Lin C.-J. (2011) LIBSVM: a library for support vector machines. ACM Trans. Intell. Syst. Technol., 2, 1–27.

[btu744-B5] Cozzetto D. Jones D.T. (2013) The contribution of intrinsic disorder prediction to the elucidation of protein function. Curr. Opin. Struct. Biol., 23, 467–472. 23466039 10.1016/j.sbi.2013.02.001

[btu744-B6] DeLong E.R. (1988) Comparing the areas under two or more correlated receiver operating characteristic curves: a nonparametric approach. Biometrics, 44, 837–845. 3203132

[btu744-B7] Deng X. (2012) A comprehensive overview of computational protein disorder prediction methods. Mol. Biosyst., 8, 114–121. 21874190 10.1039/c1mb05207a PMC3633217

[btu744-B8] Disfani F.M.*.* (2012) MoRFpred, a computational tool for sequence-based prediction and characterization of short disorder-to-order transitioning binding regions in proteins. Bioinformatics, 28, i75–i83. 22689782 10.1093/bioinformatics/bts209 PMC3371841

[btu744-B9] Dosztanyi Z.*.* (2005) The pairwise energy content estimated from amino acid composition discriminates between folded and intrinsically unstructured proteins. J. Mol. Biol., 347, 827–839. 15769473 10.1016/j.jmb.2005.01.071

[btu744-B10] Eickholt J. Cheng J. (2013) DNdisorder: predicting protein disorder using boosting and deep networks. BMC Bioinformatics, 14, 88. 23497251 10.1186/1471-2105-14-88 PMC3599628

[btu744-B11] Fang C.*.* (2013) MFSPSSMpred: identifying short disorder-to-order binding regions in disordered proteins based on contextual local evolutionary conservation. BMC Bioinformatics, 14, 300. 24093637 10.1186/1471-2105-14-300 PMC3853019

[btu744-B12] Hirose S.*.* (2007) POODLE-L: a two-level SVM prediction system for reliably predicting long disordered regions. Bioinformatics, 23, 2046–2053. 17545177 10.1093/bioinformatics/btm302

[btu744-B13] Ishida T. Kinoshita K. (2007) PrDOS: prediction of disordered protein regions from amino acid sequence. Nucleic Acids Res., 35, W460–W464. 17567614 10.1093/nar/gkm363 PMC1933209

[btu744-B14] Ishida T. Kinoshita K. (2008) Prediction of disordered regions in proteins based on the meta approach. Bioinformatics, 24, 1344–1348. 18426805 10.1093/bioinformatics/btn195

[btu744-B15] Jones D.T. Ward J.J. (2003) Prediction of disordered regions in proteins from position specific score matrices. Proteins, 53 (Suppl. 6), 573–578. 14579348 10.1002/prot.10528

[btu744-B16] Khan W.*.* (2013) Predicting binding within disordered protein regions to structurally characterised peptide-binding domains. PLoS One, 8, e72838. 24019881 10.1371/journal.pone.0072838 PMC3760854

[btu744-B17] Kozlowski L.P. Bujnicki J.M. (2012) MetaDisorder: a meta-server for the prediction of intrinsic disorder in proteins. BMC Bioinformatics, 13, 111. 22624656 10.1186/1471-2105-13-111 PMC3465245

[btu744-B18] Linding R.*.* (2003) GlobPlot: Exploring protein sequences for globularity and disorder. Nucleic Acids Res., 31, 3701–3708. 12824398 10.1093/nar/gkg519 PMC169197

[btu744-B19] Liu J. Rost B. (2003) NORSp: predictions of long regions without regular secondary structure. Nucleic Acids Res., 31, 3833–3835. 12824431 10.1093/nar/gkg515 PMC168922

[btu744-B20] Lobley A.*.* (2007) Inferring function using patterns of native disorder in proteins. PLoS Comput. Biol., 3, e162. 17722973 10.1371/journal.pcbi.0030162 PMC1950950

[btu744-B21] Melamud E. Moult J. (2003) Evaluation of disorder predictions in CASP5. Proteins, 53 (Suppl 6), 561–565. 14579346 10.1002/prot.10533

[btu744-B22] Meszaros B.*.* (2009) Prediction of protein binding regions in disordered proteins. PLoS Comput. Biol., 5, e1000376. 19412530 10.1371/journal.pcbi.1000376 PMC2671142

[btu744-B23] Minneci F.*.* (2013) FFPred 2.0: improved homology-independent prediction of gene ontology terms for eukaryotic protein sequences. PLoS One, 8, e63754. 23717476 10.1371/journal.pone.0063754 PMC3661659

[btu744-B24] Monastyrskyy B.*.* (2011) Evaluation of disorder predictions in CASP9. Proteins, 79 (Suppl 10), 107–118. 21928402 10.1002/prot.23161 PMC3212657

[btu744-B25] Monastyrskyy B.*.* (2014) Assessment of protein disorder region predictions in CASP10. Proteins, 82 (Suppl 2), 127–137. 23946100 10.1002/prot.24391 PMC4406047

[btu744-B26] Noivirt-Brik O. (2009) Assessment of disorder predictions in CASP8. Proteins, 77 (Suppl 9), 210–216. 19774619 10.1002/prot.22586

[btu744-B27] Orosz F. Ovadi J. (2011) Proteins without 3D structure: definition, detection and beyond. Bioinformatics, 27, 1449–1454. 21493654 10.1093/bioinformatics/btr175

[btu744-B28] Perkins J.R.*.* (2010) Transient protein–protein interactions: structural, functional, and network properties. Structure, 18, 1233–1243. 20947012 10.1016/j.str.2010.08.007

[btu744-B29] Prilusky J. (2005) FoldIndex: a simple tool to predict whether a given protein sequence is intrinsically unfolded. Bioinformatics, 21, 3435–3438. 15955783 10.1093/bioinformatics/bti537

[btu744-B37] R Core Team. (2012) R: A Language and Environment for Statistical Computing. R Foundation for Statistical Computing, Vienna, Austria.

[btu744-B30] Robin X.*.* (2011) pROC: an open-source package for R and S+ to analyze and compare ROC curves. BMC Bioinformatics, 12, 77. 21414208 10.1186/1471-2105-12-77 PMC3068975

[btu744-B31] Schlessinger A.*.* (2009) Improved disorder prediction by combination of orthogonal approaches. PLoS One, 4, e4433. 19209228 10.1371/journal.pone.0004433 PMC2635965

[btu744-B32] Shimizu K.*.* (2007a) POODLE-S: web application for predicting protein disorder by using physicochemical features and reduced amino acid set of a position-specific scoring matrix. Bioinformatics, 23, 2337–2338. 17599940 10.1093/bioinformatics/btm330

[btu744-B33] Shimizu K.*.* (2007b) Predicting mostly disordered proteins by using structure-unknown protein data. BMC Bioinformatics, 8, 78. 17338828 10.1186/1471-2105-8-78 PMC1838436

[btu744-B34] Sickmeier M.*.* (2007) DisProt: the database of disordered proteins. Nucleic Acids Res, 35, D786–D793. 17145717 10.1093/nar/gkl893 PMC1751543

[btu744-B35] Sillitoe I.*.* (2013) New functional families (FunFams) in CATH to improve the mapping of conserved functional sites to 3D structures. Nucleic Acids Res., 41, D490–D498. 23203873 10.1093/nar/gks1211 PMC3531114

[btu744-B36] Suzek B.E.*.* (2007) UniRef: comprehensive and non-redundant UniProt reference clusters. Bioinformatics, 23, 1282–1288. 17379688 10.1093/bioinformatics/btm098

[btu744-B38] UniProt Consortium. (2014) Activities at the Universal Protein Resource (UniProt). Nucleic Acids Res., 42, D191–D198. 24253303 10.1093/nar/gkt1140 PMC3965022

[btu744-B39] Velankar S.*.* (2013) SIFTS: Structure Integration with Function, Taxonomy and Sequences resource. Nucleic Acids Res, 41, D483–D489. 23203869 10.1093/nar/gks1258 PMC3531078

[btu744-B40] Wang G. Dunbrack R.L. Jr (2005) PISCES: recent improvements to a PDB sequence culling server. Nucleic Acids Res., 33, W94–W98. 15980589 10.1093/nar/gki402 PMC1160163

[btu744-B41] Wang X.*.* (2010) A large intrinsically disordered region in SKIP and its disorder-order transition induced by PPIL1 binding revealed by NMR. J. Biol. Chem., 285, 4951–4963. 20007319 10.1074/jbc.M109.087528 PMC2836099

[btu744-B42] Ward J.J.*.* (2004) Prediction and functional analysis of native disorder in proteins from the three kingdoms of life. J. Mol. Biol., 337, 635–645. 15019783 10.1016/j.jmb.2004.02.002

[btu744-B43] Zemla A. (2003) LGA: a method for finding 3D similarities in protein structures. Nucleic Acids Res., 31, 3370–3374. 12824330 10.1093/nar/gkg571 PMC168977

