# Supplementary material for: DISOPRED3: precise disordered region predictions with annotated protein-binding activity
Source: Bioinformatics. 2014 Nov 12;31(6):857–63. doi: 10.1093/bioinformatics/btu744 (PMC4380029; doi:10.1093/bioinformatics/btu744)
Supplement: Supplementary Data [file supp_31_6_857__index.html]

DISOPRED3: Precise disordered region predictions with annotated protein binding activity — DISOPRED3: precise disordered region predictions with annotated protein-binding activity — DISOPRED3: precise disordered region predictions with annotated protein-binding activity — Supplementary Data 

# DISOPRED3: precise disordered region predictions with annotated protein-binding activity

## Supplementary Data

files

**Files in this Data Supplement:**

- Supplementary Data - docx file
