# Supplementary material for: DISOPRED3: precise disordered region predictions with annotated protein-binding activity
Source: Bioinformatics. 2014 Nov 12;31(6):857–63. doi: 10.1093/bioinformatics/btu744 (PMC4380029; doi:10.1093/bioinformatics/btu744)
Supplement: Supplementary Data [file supp_btu744_supplementary_data.docx]

**Supplementary Table 1.** List of known disordered protein regions and disordered protein binding sites therein used in this study.

| **UniProtKB** | **Disprot** | **PDB chain** | **known disordered regions** | **disordered protein binding sites** | **PubMed identifier** |
| --- | --- | --- | --- | --- | --- |
| O43236-6 | NA | NA | 248-274 | 267-274 | *21949740* |
| P01094 | DP00179 | 1dpjB | 1-68 | 2-34 | 10655612 |
| P01730 | DP00123 | 3b71D | 421-458 | 431-441 | 9622505; 18078954 |
| P02686-5 | DP00236 | 1ymmC | 1-171 | 84-108 | 7500383; 15821740 |
| P02686-6 | DP00237 | NA | 1-171 | 146-166 | 22405011 |
| P04637 | DP00086 | 1dt7X | 367-388 | 367-388 | 10876243 |
| P04638 | DP00087 | 1ycqB | 1-73 | 17-27 | 8875929 |
| P04639 | DP00088 | 2b3gB | 1-73 | 33-56 | 16234232 |
| P05221 | DP00217 | 1ee5B | 120-200 | 153-171 | 10745017 |
| P07766 | DP00506 | 1a81B | 153-207 | 186-203 | 14967045; 9698567 |
| P09883 | DP00342 | 3o0eL; 2ivzE | 1-83 | 2-16; 32-47 | 21098297; 16894158 |
| P45976 | DP00625 | 3c66C | 1-220 | 81-85; 92-97 | 18537269 |
| P46108 | DP00748 | 1ju5A | 1-9; 65-85; 121-133; 192-304 | 65-85 | 12384576 |
| P46937 | DP00702 | 3kisB | 61-100 | 86-100 | 20123905 |
| P60896 | DP00617 | 1miuB | 1-70 | 7-25; 37-63 | 12228710 |
| P9WHN5 | DP00293 | 3m91B | 1-64 | 21-51 | 19580545; 20953180 |
| Q06253 | DP00288 | 3dd7B | 1-73 | 52-73 | 9915794; 18757857 |
| Q13573 | DP00608 | NA | 1-172 | 59-79 | 20007319 |
| Q6BBK3 | NA | 3b1jC | 1-75 | 51-75 | 22153507 |
| Q98XH7 | NA | 3o6lC | 1-72 | 2-16 | 16423825; 21035463 |
| Q99LM3 | DP00742 | 1jv9A | 1-346; 449-459 | 449-459 | 18477568 |
| Q9Y3M2 | DP00709 | NA | 1-63 | 16-22; 21-29 | 21182262; 19940019 |
| Q9Y6Q9-3 | DP00343 | 1kbhA | 1023-1093 | 1045-1091 | 11823864 |

Rows shaded in light blue correspond to proteins used for comparison with other tools.

**Supplementary Table 2.** Comparison of DISOPRED3 and DISOPRED2 performance divided by IDR length ranges in the CASP9 dataset.

| Subste | Method | Sensitivity | Specificity | Precision | MCC | AUC |
| --- | --- | --- | --- | --- | --- | --- |
| No IDR shorter than 4 aas | DISOPRED2 | 0.343 | 0.952 | 0.422 | 0.324 | 0.733 |
|  | DISOPRED3 | 0.347 | 0.992 | 0.823 | 0.508 | 0.854 |
| No IDR shorter than 20 aas | DISOPRED2 | 0.264 | 0.952 | 0.192 | 0.186 | 0.681 |
|  | DISOPRED3 | 0.432 | 0.992 | 0.711 | 0.540 | 0.871 |
| No IDR shorter than 30 aas | DISOPRED2 | 0.170 | 0.952 | 0.070 | 0.079 | 0.622 |
|  | DISOPRED3 | 0.436 | 0.992 | 0.548 | 0.479 | 0.831 |
| No IDR shorter than 40 aas | DISOPRED2 | 0.147 | 0.952 | 0.027 | 0.043 | 0.539 |
|  | DISOPRED3 | 0.265 | 0.992 | 0.237 | 0.244 | 0.736 |

**Supplementary Table 3.** Performance comparison between DISOPRED releases by IDR position along CASP target sequences.

| Subset | Method | Sensitivity | Specificity | Precision | MCC | AUC |
| --- | --- | --- | --- | --- | --- | --- |
| Terminal protein regions | DISOPRED2 | 0.604 | 0.762 | 0.615 | 0.367 | 0.749 |
|  | DISOPRED3 | 0.646 | 0.914 | 0.825 | 0.594 | 0.868 |
| Internal protein regions | DISOPRED2 | 0.199 | 0.964 | 0.278 | 0.190 | 0.688 |
|  | DISOPRED3 | 0.181 | 0.997 | 0.820 | 0.369 | 0.807 |

Terminal protein regions consist of the 10 amino acids closest to the target sequence termini; internal positions are all the remaining ones.

**Supplementary Table 4.** DISOPRED3 IDR prediction accuracy on the test protein chains used for comparison with tools predicting disordered regions folding upon protein binding..

| **UniProtKB** | **TP** | **FP** | **FN** | **TN** | **Sensitivity** | **Specificity** | **Precision** | **F1** | **MCC** |
| --- | --- | --- | --- | --- | --- | --- | --- | --- | --- |
| O43236-6 | 14 | 84 | 13 | 163 | 0.519 | 0.660 | 0.143 | 0.224 | 0.111 |
| P45976 | 159 | 92 | 61 | 15 | 0.723 | 0.140 | 0.633 | 0.675 | -0.152 |
| P46108 | 13 | 0 | 143 | 148 | 0.083 | 1.000 | 1.000 | 0.154 | 0.206 |
| P46937 | 39 | 150 | 1 | 314 | 0.975 | 0.677 | 0.206 | 0.341 | 0.364 |
| P60896 | 6 | 0 | 64 | 0 | 0.086 | NA | 1.000 | 0.158 | NA |
| Q06253 | 1 | 0 | 72 | 0 | 0.014 | NA | 1.000 | 0.027 | NA |
| Q98XH7 | 26 | 0 | 46 | 0 | 0.361 | NA | 1.000 | 0.531 | NA |
| Q99LM3 | 230 | 0 | 127 | 102 | 0.644 | 1.000 | 1.000 | 0.784 | 0.536 |
| Q9Y3M2 | 21 | 6 | 42 | 57 | 0.333 | 0.905 | 0.778 | 0.467 | 0.290 |

**Supplementary Table 5.** Number and percentage of false positive assignments of disordered protein binding residues that different classifiers make as a function of their sequence separation from the validated sites.

| **Method** | **Maximum sequence separation** | **Number of false positives** | **Percentage of false positive** |
| --- | --- | --- | --- |
| ANCHOR | 5 | 29 | 3.06 |
|  | 10 | 46 | 4.85 |
|  | 20 | 87 | 9.17 |
|  | 35 | 139 | 14.65 |
| DISOPRED3 | 5 | 14 | 16.28 |
|  | 10 | 21 | 24.42 |
|  | 20 | 24 | 27.91 |
|  | 35 | 25 | 29.07 |
| MoRFpred | 5 | 3 | 1.88 |
|  | 10 | 7 | 4.38 |
|  | 20 | 16 | 10.00 |
|  | 35 | 16 | 10.00 |
| MSFPSSMpred | 5 | 10 | 5.62 |
|  | 10 | 10 | 5.62 |
|  | 20 | 22 | 12.36 |
|  | 35 | 32 | 17.98 |
